# Study on the Reutilization of Clear Fracturing Flowback Fluids in Surfactant Flooding with Additives for Enhanced Oil Recovery (EOR)

**DOI:** 10.1371/journal.pone.0113723

**Published:** 2014-11-19

**Authors:** Caili Dai, Kai Wang, Yifei Liu, Jichao Fang, Mingwei Zhao

**Affiliations:** 1 China University of Petroleum (East China), Qingdao, Shandong, 266580, People’s Republic of China; 2 China National Offshore Oil Corporation Research Institute, Beijing, 100028, People’s Republic of China; China University of Mining and Technology, China

## Abstract

An investigation was conducted to study the reutilization of clear fracturing flowback fluids composed of viscoelastic surfactants (VES) with additives in surfactant flooding, making the process more efficient and cost-effective. The clear fracturing flowback fluids were used as surfactant flooding system with the addition of α-olefin sulfonate (AOS) for enhanced oil recovery (EOR). The interfacial activity, emulsification activity and oil recovery capability of the recycling system were studied. The interfacial tension (IFT) between recycling system and oil can be reduced by 2 orders of magnitude to 10^−3^ mN/m, which satisfies the basic demand of surfactant flooding. The oil can be emulsified and dispersed more easily due to the synergetic effect of VES and AOS. The oil-wet surface of quartz can be easily converted to water-wet through adsorption of surfactants (VES/AOS) on the surface. Thirteen core plug flooding tests were conducted to investigate the effects of AOS concentrations, slug sizes and slug types of the recycling system on the incremental oil recovery. The investigations prove that reclaiming clear fracturing flowback fluids after fracturing operation and reuse it in surfactant flooding might have less impact on environment and be more economical.

## Introduction

Hydraulic fracturing [1] is the most effective technique to enhanced oil recovery (EOR), which plays an important role in ultra-low permeable oilfield development all over the world [2]–[4]. During the fracturing process, hydraulic fracturing fluids are injected into the formation to create the required fractures which results in the optimum performance of oil wells. The development of fracturing fluids has been separated into three stages: active water, gelled hydrocarbons and transition metal cross-linked guar-based fluids [5]. Although these fracturing fluids are used most widely, their disadvantages seriously limit their extensive applications, such as poor sand carrying capability and great damage to the formation [6]. In recent years, a polymer-free clear fracturing fluid has been firstly proposed by Schlumberger as the substitute of polymer-based fracturing fluids with characteristics of anti-shearing, low friction, easy gel breaking without gel breaker and low formation damage [7]–[9]. Cristian Fontana et al. performed rheological and conductivity test of viscoelastic surfactant (VES) and polymeric fluid respectively [10]. The results showed that their rheological properties were similar, however, the retained conductivity of VES was 77% and that of polymeric fluid was 44%, indicating its lower formation damage. P. F. Sullivan et al. studied the VES and low-guar fluid applications in two identical wells at the Mesaverda reservoir in Rock Spring, Wyoming [11], [12]. The results showed that the friction pressure associated with VES treatment was 30∼50% lower than low-guar treatment while maintaining a relative high oil production even after 9 months.

Although the hydraulic fracturing process is widely used to improve oil production, there still exists a serious problem for all kinds of fracturing fluids. How to deal with fracturing flowback fluids whose volumes for a fracturing treatment are very huge and generally range from a few thousands gallons to several hundred thousands gallons, is a great challenge [13]. What’s more, fracturing flowback fluids contain hazardous materials, such as methanal, oil in water emulsion and various kinds of additives. If these flowback fluids cannot be correctly treated, they may cause serious environmental pollution.

In order to avoid such issues, the reutilization of clear fracturing flowback fluids is regarded as the best method, which mainly has three benefits: the cost savings through recovering and reusing chemicals [14], the cost savings of clean water resources for subsequent treatment, and the elimination of disposal costs.

Up to now, most of the researches reuse fracturing flowback fluids in secondary fracturing process. Zhanqing Qu et al. proposed reutilization of guar based fracturing fluid in fracturing process by adjusting pH value of flowback fluids [15]. The viscosities of initial and reusing system being sheared for 90 min are 200 mPa·s^−1^ and 85 mPa·s^−1^, respectively, which are both suitable for fracturing. Qianding Li et al [16] studied the reutilization of hydroxypropyl guar fracturing fluid through adding fresh cross linking agent and thickener into flowback fluids to conduct secondary fracturing process.

Surfactant-based chemical-flooding process has been widely studied for its advantage on high oil displacement efficiency, and many studies have been conducted [17]–[20]. These studies concentrated on three main mechanisms for EOR: (1) the capability of surfactants to reduce interfacial tension to ultra-low level between aqueous phase and residual oil [21], [22]. (2) wettability alteration by adsorption of surfactants on the reservoir rock [23]–[25]. (3) emulsification-entrainment, emulsification-entrapment to improve the sweep efficiency [26].

As is known, the clear fracturing fluids are made up of a variety of viscoelastic surfactants (VES), such as anionic surfactants, cationic surfactants and zwitterionic surfactants [27]. Furthermore, the amount of VES in clear fracturing flowback fluids is considerably large after a fracturing stimulation for its huge consumption of several thousands of gallons and high application concentration from 3 wt% to 8 wt% [28], [29]. This inspires us to study whether the clear fracturing flowback fluids can be reused in surfactant flooding for EOR. In this work, we study the reutilization of clear fracturing flowback fluids in surfactant flooding. To the best knowledge of our known, this may be the first time to reuse clear fracturing flowback fluids in surfactant flooding for EOR. Through this work, we expect to realize the reutilization of clear flowback fluids in surfactant flooding and to provide solid foundation for better reusing clear fracturing flowback fluids efficiently and cost-effectively.

## Materials and Methods

### Materials

The chemicals used in this study include VES (the main component of clear fracturing fluid, a quaternary ammonium surfactant, Changqing oilfield, China), α-olefin sulfonate (AOS, Shengli oilfield, China), sodium chloride and calcium chloride anhydrous (Sinopharm Chemical Reagent Co. Ltd).

The oil was collected from Ansaichang reservoir (Changqing oilfield, China). The viscosity of oil was 2.2 mPa·s^−1^ at 61°C (the reservoir temperature) as measured by a Brookfield DV-II viscometer. The density of oil was 767 kg/m^3^. Oil was separated from water and solids by centrifugation.

The formation water was used in the experiments. The formation water was treated to eliminate the solids and floating oil droplets before experiment. The compositional analysis of formation water is shown in Table 1.

**Table 1:** Quality analysis of formation water.

ions	K^+^+Na^+^	Ca^2+^	Mg^2+^	Cl^−^	SO_4_^2−^	HCO_3_^−^
concentration (mg/L)	20684	7977	909	48130	544	184
total salinity (mg/L)			78428			

### Preparation of clear fracturing flowback fluids in laboratory

In laboratory, clear fracturing flowback fluids was obtained by simulating the actual gelling and gel breaking process during fracturing construction in the field. The gelling fluid was prepared with 4 wt% VES and 96 wt% formation water. The gel breaking process was realized by adding 2 wt% kerosene into the gelling system. Then, it was placed under reservoir temperature in a separating funnel until the viscosity of system reduces to that of brine. The supernatant obtained in separating funnel was the clear fracturing flowback fluids and would be used for the following experiments.

### Interfacial tension measurement

The interfacial tension (IFT) between oil and water was measured by the Texas-500 spinning drop tension meter based on the following equation [30]. The interfacial tension was determined with a single-measurement method and all measurements were repeated at least twice.
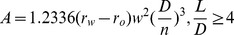



Where *A* is the interfacial tension between oil and water (mN/m); *r*_w_ and *r*_o_ are the density of the water phase and oil phase (g/cm^3^); *w* is the rotational velocity (rpm); *D* and *L* are the width and length of the oil droplet (mm); *n* is the refractive index of the water phase.

### Surface tension measurement

Surface tension measurements were carried out on a Model JYW-200B surface tension meter (Chengde Dahua Instrument Co.Ltd., accuracy ±0.01 mN/m) using the ring method. Temperature was controlled by thermostat cell holder. The surface tension was determined with a single-measurement method and all measurements were repeated at least twice.

### Porosity measurement

The porosity was determined gravimetrically by the following procedure;

(1) The volume of artificial cores, V was calculated; (2) the dry weight of the artificial cores, m1 was determined; (3) the artificial cores were saturated by circulating the formation water of density ρ with a pump (SHZ-DIII, Shanghai Cheng Ming Instrument Equipment Co. Ltd. China) for 24 hours, (The saturation was assumed to be complete when bubbles were not produced) (4) the wet weight of the artificial cores, m2 was determined; (5) the porosity was calculated using the following relationship;
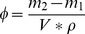



Where *Φ* is the porosity; *m_1_* and *m_2_* respectively are the dry-weight and the wet-weight of artificial cores; *V* is the volume of artificial cores; *ρ* is the density of formation brine.

### Emulsification test

Under laboratory conditions, the emulsions were prepared by mechanical emulsion method. The chemical agents were mixed completely with formation water and then mixing with crude oil by using electric mulser (SFS-S400, Siehe China Corp) at a stirring rate of 3000 rpm for 10 min at 61°C. The ratio of oil/water was 1∶1.

Photomicrographs of emulsions were taken by using a microscope (XSP-8CE, Changfang Corp, China). For emulsion stability tests, emulsions were put into graduated test tube with stopper, which could easily cause the separation of oil/water phase fluids because of their density difference. The emulsifying stability is characterized by water separation rate that is the ratio of accumulated separated water volume to the total water volume. The formula is shown as following.
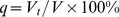



Where, *q* is water separation rate, %; *V_t_* is the volume of accumulated separated water, mL; *V* is the total water volume involved in emulsion, mL.

### Wettability test

**Figure 1:**
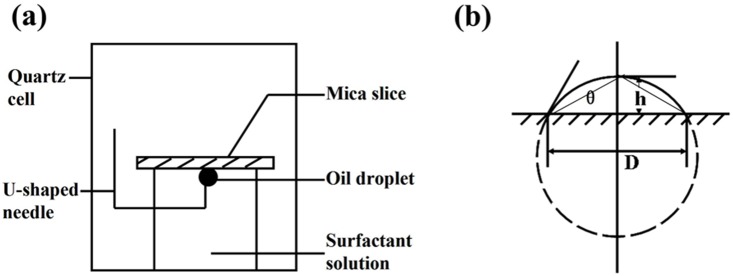
Principle of wettability measurement. (a) The schematic diagram of the contact angle measurement system; (b) The schematic diagram of measuring the contact angle.

The wettability of the quartz plate was evaluated by static contact angle. The flowchart and mechanism of the optical projection for measuring the static contact angle is shown in Figure 1.

Firstly, the quartz plates (25×25×2 mm) was saturated in chromic acid solution for 24 h and then washed by deionized water. The plates were treated in the three following ways: dried, soaked in deionized water and formation water successively for 24 h to simulate the formation of initial reservoir [31]. Secondly, the wet slides taken out from water or salt solutions were put into vacuum drying oven (DZF-1 Botai China Corp) to remove the water and then immediately transferred into crude oil mixed with 20 v% n-heptane to age for 24 h under 61°C. Then the plates were treated with n-heptane to get rid of spare crude oil on the plates’ surface. Thirdly, put these plates into surfactant solutions of different concentrations to age for 24 h under 61°C and then dried them out following step 2. Finally, the static contact angle measurements were performed at room temperature (25°C) using a Kruss Contact Angle Meter DSA100 quartz cell as shown in Figure 1 [32]. The average value of at least three consecutive experiments was taken for each point. The formula is shown as following.
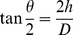



Where, *θ* is the wetting angle of oil phase °; *h* is the height of oil droplet on the projection, mm; *D* is the length of oil droplet bottom on the projection, mm;

### Core plug flooding test

Core holder of 2.54 cm in diameter and 30 cm in length (Haianxian Oil Scientific Research Apparatus Co Ltd, China) was used for the core plug flooding test. Core plugs (Haianxian Oil Scientific Research Apparatus Co Ltd, China) with permeability ranging from 2 mD to 4 mD were used to simulate actual conditions of Ansaichang reservoirs.

Following procedure was followed in conducting the core plug flooding test; (1) The permeability of the core plugs was measured with formation water; (2) saturating cores with crude oil until no more water was produced from the outlet; (3) water flooding cores until no more oil was produced from the outlet (water cut>98%); (4) the recycling system slugs were injected; and (5) the subsequent water flooding was conducted until oil production became negligible (water cut>98%). All of the tests were conducted at 61°C, and the injection flow rate was 0.1 mL/min.

In this test, a total of 13 core plug flooding tests were conducted. The parameters of these artificial cores are shown in Table 2. The parameters of different recycling system slugs in test are shown in Table 3. Each core plug flooding test was repeated at least twice.

**Table 2:** Summary of core plug parameters.

core plugparameter	Unit	No. 1	No. 2	No. 3	No. 4	No. 5	No. 6	No. 7	No. 8	No. 9	No. 10	No. 11	No. 12	No. 13
porosity	V %	15.6	16.2	17.3	16.8	16.3	15.5	15.9	18.3	16.5	17.4	17.6	16.5	15.8
diameter	cm	2.5	2.5	2.5	2.5	2.5	2.5	2.5	2.5	2.5	2.5	2.5	2.5	2.5
length	cm	10.0	10.0	10.0	10.0	10.0	10.0	10.0	10.0	10.0	10.0	10.0	10.0	10.0
pressuredrop	MPa	0.36	0.46	0.63	0.32	0.60	0.68	0.51	0.40	0.31	0.29	0.60	0.44	0.38
waterpermeability	mD	2.8	2.2	1.6	3.2	1.7	1.5	2.0	2.5	3.3	3.5	1.7	2.3	2.7
original oilsaturation	OOIP%	73.0	72.0	67.7	75.0	72.2	65.8	74.9	76.7	79.3	77.5	74.5	69.8	72.3
water floodingrecovery	OOIP%	52.5	62.4	61.2	62.6	55.8	56.7	52.0	55.2	57.6	56.5	56.7	54.3	58.9

**Table 3:** Summary of chemical slugs in core plug flooding test (61°C).

core plug no.	chemical agents (wt%)	slug size (PV)
1	0.04 wt% AOS	0.50
2	0.2 wt% VES	0.50
3	0.01 wt% AOS+0.2 wt% VES	0.50
4	0.02 wt% AOS+0.2 wt% VES	0.50
5	0.04 wt% AOS+0.2 wt% VES	0.50
6	0.06 wt% AOS+0.2 wt% VES	0.50
7	0.08 wt% AOS+0.2 wt% VES	0.50
8	0.04 wt% AOS+0.2 wt% VES	0.10
9	0.04 wt% AOS+0.2 wt% VES	0.30
10	0.04 wt% AOS+0.2 wt% VES	0.70
11	0.04 wt% AOS+0.2 wt% VES	0.90
12	0.2 wt% VES, 0.04 wt% AOS	0.25,0.25
13	0.04 wt% AOS, 0.2 wt% VES	0.25,0.25

## Results and Discussion

### Minimum interfacial tension behavior of oil/brine/clear fracturing flowback fluids and oil/brine/recycling system

In surfactant or alkaline flooding systems, capillary number [33] is a critical dimensionless number to the residual oil saturation of reservoir as shown in Figure S1 in File S1. It is defined as the ratio of viscous force to capillary force of displacing phase and its formula is shown as follows:
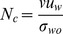



Where, *N*_c_ is capillary number; *v* is displacement velocity (m/s); *σ* is the oil/water interfacial tension (mN/m); *u*_w_ is viscosity of displacing phase (mPa/s).

As is known, the increase of capillary number to a certain magnitudes contributes a lot to the reduction of residual oil saturation, which can reach as low as 20%. The capillary number formula shows that the *N*_c_ is significantly increased if the oil/water IFT can be reduced by 2 or 3 orders of magnitude [34]. So, the reduction of IFT as low as possible is very important to the final oil recovery, which is generally a decisive factor in the surfactant flooding.

**Figure 2:**
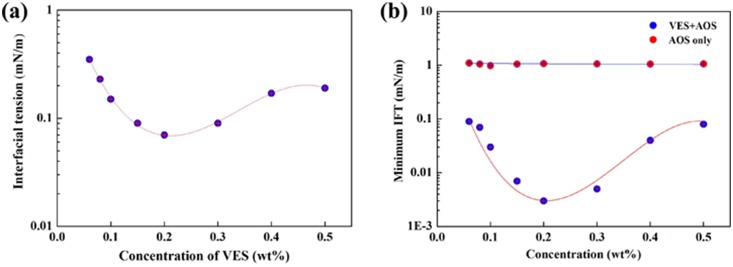
Interfacial tension plots. (a) IFT plots between clear fracturing flowback fluids and oil with different VES concentrations; (b) IFT plots between recycling system and oil as a function of different VES concentrations for 0.04 wt% AOS.

From Figure 2 (a), a series of interfacial tension tests were carried out to investigate the ability of fracturing flowback fluids to reduce oil/water interfacial tension. Figure 2 (a) shows IFT between fracturing flowback fluid and oil *versus* concentrations at 61°C (the reservoir temperature). With the increase of surfactant concentration, the IFT decreases rapidly and reaches a minimum, which indicates the saturation of adsorption of surfactants [35] on oil/water interface. With the further increase of surfactant concentration, the IFT increases gradually, which may be due to the solubilization of surfactants by micelles formed in aqueous phase. According to the IFT values, the ability of decreasing IFT is best at the VES concentration of 0.2 wt%. However, the lowest IFT is only about 10^−1^ mN/m, which can not satisfy the demand of surfactant flooding.

**Figure 3:**
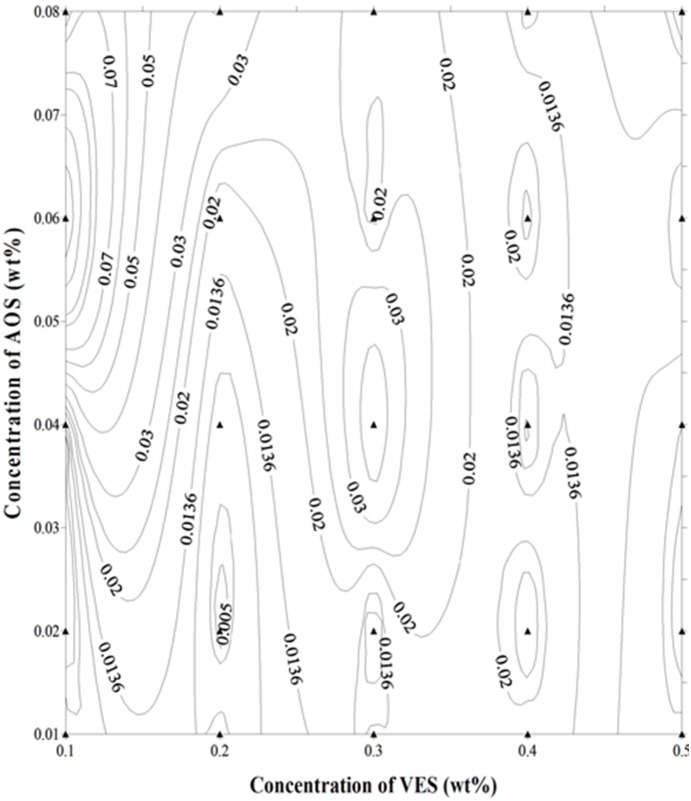
Contour map of interfacial tension between VES/AOS system and crude oil.

To further decrease the IFT between oil and flowback fluid, another surface active agent AOS is added. The IFT curves between oil and AOS solutions with different concentrations were shown in Figure 2 (b). The IFT between AOS aqueous solution/oil around 10^0^ mN/m with its concentration ranging from 0.06 wt%∼0.5 wt%, indicating that it contributes little to the reduction of IFT. Although the ability of AOS itself on decreasing IFT is limited, it contributes a lot to the recycling system with little dosages as shown in Figure 2 (b). Compared with the IFT between single VES and oil, the IFT of VES/AOS solution and oil can be reduced rapidly to 3×10^−3^ mN/m when the AOS concentration is 0.04 wt%, which can satisfy the demand of surfactant flooding. From the contour shown in Figure 3, the addition of an extremely low concentration of AOS ranging from 0.01∼0.08 wt% to clear fracturing flowback fluids has obvious effect on decreasing the IFT. Such a low amount of AOS is very efficient and cost-effective. The high effectiveness of the mixture to decrease IFT may be due to the synergistic effect of VES and AOS. As mentioned above, the clear fracturing flowback fluid is mainly composed of VES which is usually cationic ammonium surfactant and AOS is anionic. When cationic and anionic surfactants are used together, the strong electrostatic attraction leads to the adsorption of more surfactant molecules on the oil/water interface [36], which results in the appearance of lowest IFT in mixed catanionic systems.

### Surface tension test

**Figure 4:**
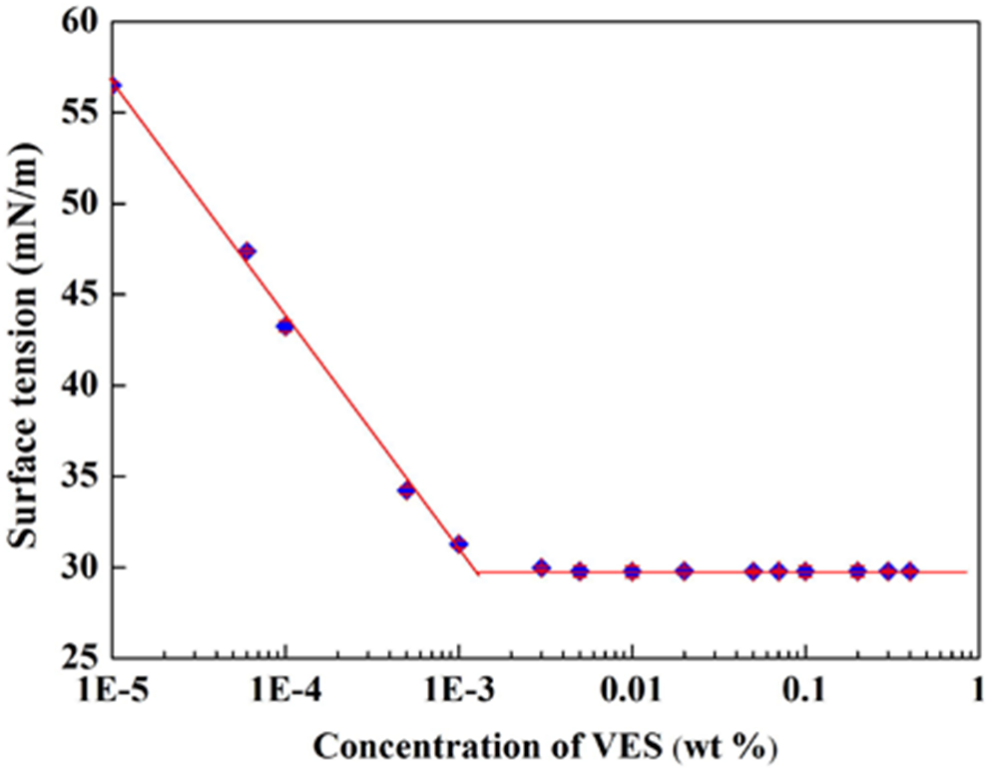
Surface tension isotherms at 61°C as a function of VES/AOS concentration.

Surface tension measurement was conducted to study properties of recycling system. Figure 4 shows surface tensions *versus* concentrations at 61°C. In the dilute solutions, the surface tension decreases sharply with the increase of VES concentrations, which indicates the adsorption of surfactants on the air/water surface. With the further increase of VES concentrations, the surface tension slowly decreases. Until surfactant concentration reaches a critical value, the surface tension no longer decreases and almost remains constant, which suggests the adsorption of surfactant molecules at surface achieves saturation. The concentration of the breakpoint in Figure 4 is traditionally assigned to be the critical aggregation concentration (cac). With continuous increase of surfactant concentrations, the micelles are formed in aqueous phase. From Figure 4, the cac values in aqueous solution at 61°C are 0.0005 wt%, which shows a relatively low cac compared with conventional surfactant [37]. So the recycling system has very good surface activity.

### Emulsification behavior of oil/brine/recycling system

Emulsification is a significant index in chemical flooding processes, particularly in surfactant flooding. In the meantime, it is also one of the important mechanisms in enhanced oil recovery (EOR) [38]. To assess the effectiveness of the recycling system in emulsifying the oil, numbers of emulsification tests were carried out to investigate their stability by the method described above.

**Figure 5:**
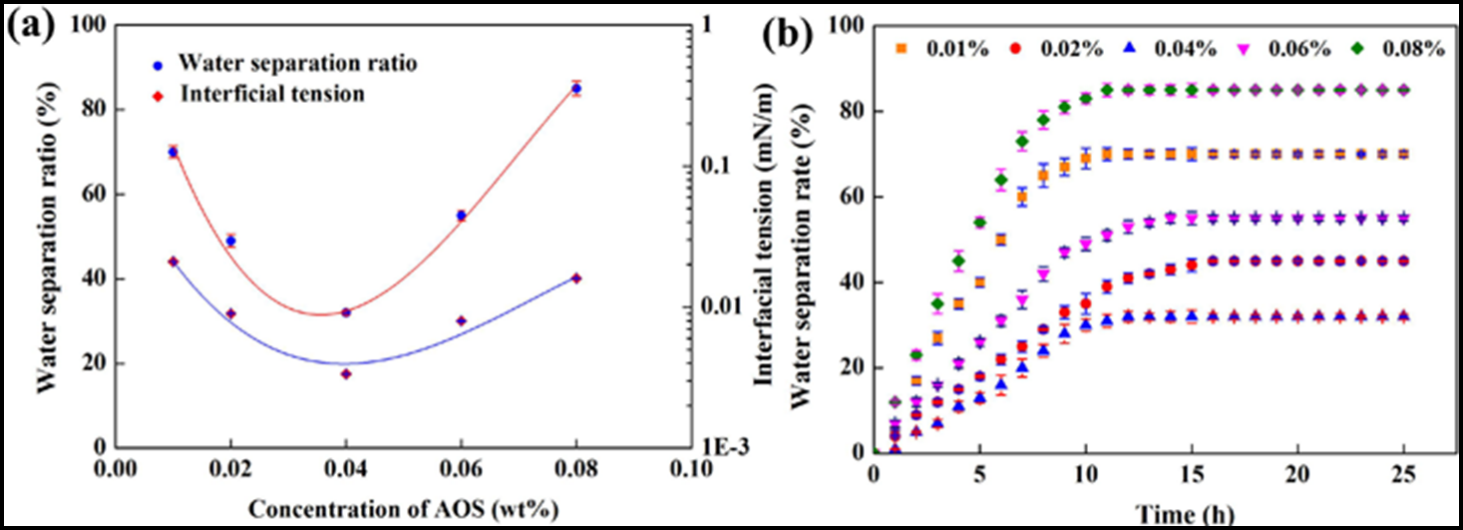
Interfacial tension and water separation ratio plots. (a) Relationship between interfacial tension and water separation ratio as a function of different AOS concentrations for the 0.2 wt% VES; (b) Relationship between water separation ratio and time as a function of different AOS concentrations for the 0.2 wt% VES.

Figure 5 (a) shows the relationship between emulsification stability and interfacial tension with variety of AOS concentration, and the VES concentration is 0.2 wt%. It indicates that the water separation ratio and minimum IFT both decrease with the increase of AOS concentrations from 0.01 to 0.04 wt%. With further increase of AOS concentration, the water separation ratio and IFT both relatively increase. Figure 5 (b) shows the relationship between water separation ratio and standing time, and indicates its excellent emulsification stability against time. During the forming of emulsions, a phenomenon was observed: the lower the IFT, the easier the emulsification occurs. In addition, Figure 5 (a) and (b) shows that the emulsions formed by recycling system with lowest IFT is more stable than other systems. This result can be found in the studies researched by Rudin et al [39]. Kokal. Rudin et al. [40] found that the lower the IFT, the more stable of emulsions formed in the emulsification process. Kakal thought that the emulsion was stabilized by surfactant, which tended to adsorb at the oil/water interface and led to a low interfacial tension.

**Figure 6:**
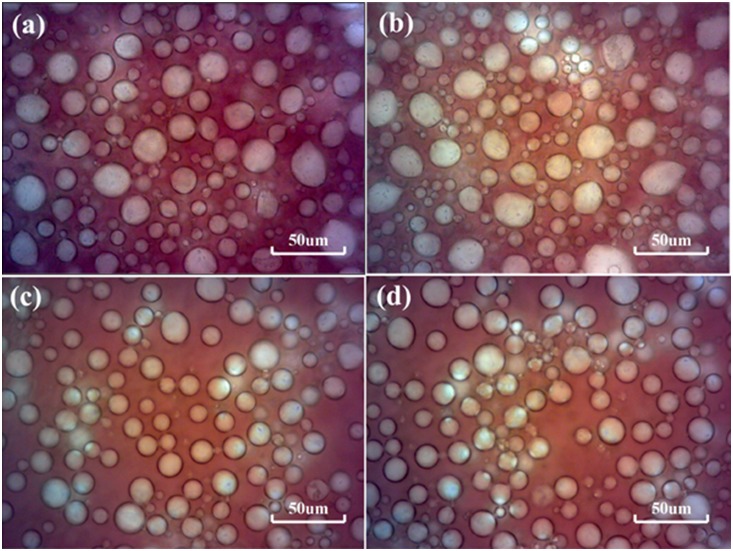
Microscope images. Micrographs of water-in oil emulsions at 400× magnification with different chemical agents, (a) and (b) 0.2 wt% VES; (c) and (d) 0.2 wt% VES+0.04 wt% AOS;

Photomicrographs of two emulsions prepared by stirring oil with different chemical solutions (emulsion a and b: 0.2 wt% VES; emulsion c and d: 0.2 wt% VES+0.04 wt% AOS) are shown in Figure 6. It is found that the transparent water phase is dispersed water droplet, which indicates that emulsions are water in oil type. The particles size, particles shape and distribution of emulsified water droplets in emulsion c and d are more homogeneous than those of emulsion a and b. Because the oil/water interfacial tension can be reduced to a lower degree in emulsion c and d, indicating that a lower interfacial tension plays a significant role in the forming of emulsions. It is consistent with the macroscopical results of water separation ratio and the analysis above.

### Wettability Test

According to Craig [41], wettability is defined as “the tendency of one fluid to spread on or adhere to a solid surface in presence of other immiscible fluids”. In the surfactant flooding process, wettability alteration of reservoir rocks is significant as it controls the multiphase flow issues including the oil migration from source rocks to primary production, capillary pressure, imbibitions, drainage, dispersion, irreducible water saturation, residual oil saturation, and EOR.

**Figure 7:**
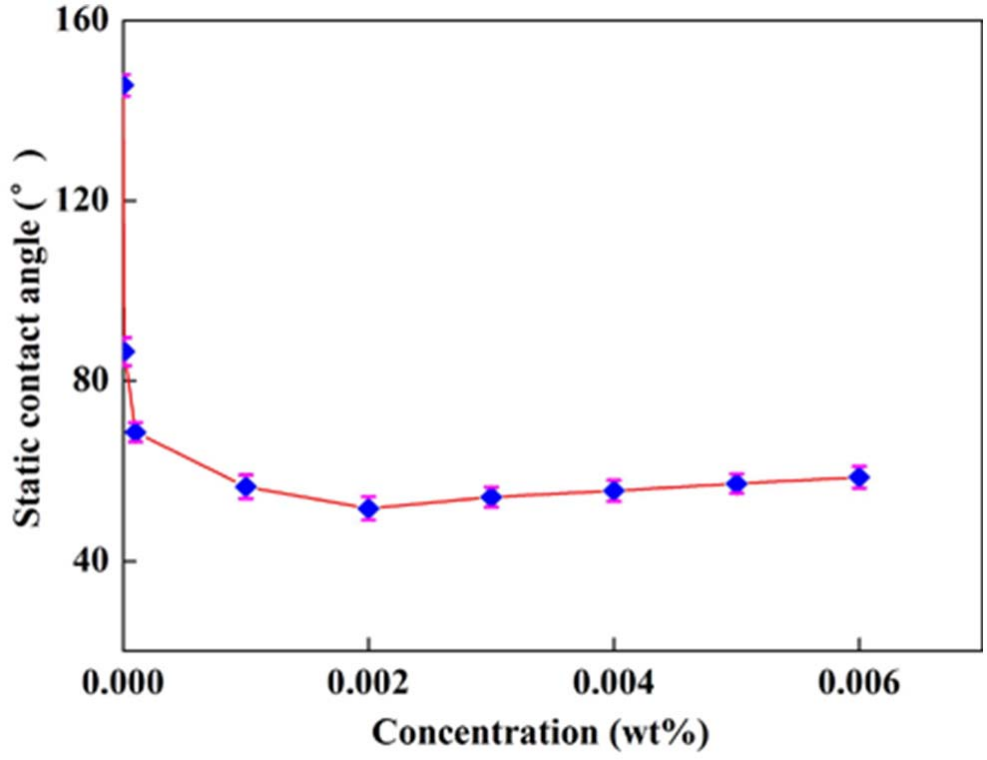
Effect of VES concentrations on contact angle with AOS concentration fixed at 0.02 wt%.

Surfactant generally consists of a hydrophobic chain and a hydrophilic group. The amphiphilicity of surfactants makes them form a multitude of different structures in solution and adsorb at interfaces [42] as shown in Figure 7. Figure 7 illustrates that with the increase of surfactant, their adsorptions on the surface results in the alteration of wettability. At low concentration smaller than 0.002 wt%, it is a stage of adsorption and the absorbing capacity increases over concentration, resulting in the reduction of static contact angel as shown in Figure 7. At the concentration of 0.002 wt%, the surfactant molecules are saturated and packed closely on the surface of quartz plate that form a closest adsorption layer, and results in a greatest change of static contact angel. With a further increase of concentration, the quartz surface starts a stage of double-layer adsorption which causes weakness of hydrophilicity of it [43]. The static contact angel changes from 145.6° to 58.2° indicating the better performance of recycling system on wettability alteration.

### Core plug flooding test

The core plug flooding test is used broadly in laboratory to assess the efficiency of chemical flooding systems. Thirteen core plug flooding tests were conducted to evaluate the effectiveness of recycling system for EOR through methods described above. The effects of AOS concentration, recycling system slug size, and recycling system slug type on the oil recovery were studied in this work.

#### Effect of AOS concentration

To research the effect of concentrations of AOS in the recycling system (VES concentration is 0.2 wt%) for EOR, five core plug flooding tests (No. 3 to 7) were conducted with AOS concentration varying from 0.01 to 0.1 wt%. All the slug size injected is 0.5 PV (pore volume) during these tests. The parameter incremental oil recovery is used to estimate the effect of AOS concentration on EOR.

**Figure 8:**
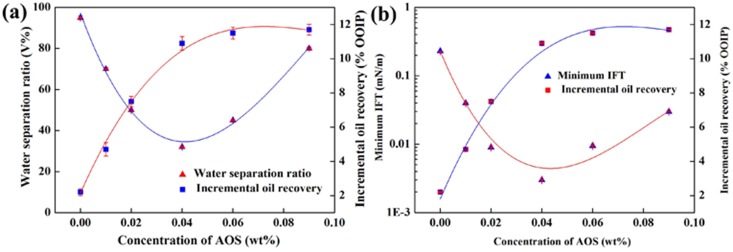
Effect of interfacial tension and emulsification on incremental oil recovery. (a) Relationship between water separation ratio and incremental oil recovery when AOS concentration varies. The VES concentration is 0.2 wt%; (b) relationship between minimum IFT and incremental oil recovery when AOS concentration varies. The VES concentration is 0.2 wt%.

Figure 8 (a) shows that, when the AOS concentration is ranging from 0 to 0.04 wt%, the incremental recovery increases rapidly from 2.2 OOIP% to 10.9 OOIP%. The result indicates that, with an appropriate AOS concentration added into the clear fracturing flowback fluids, the oil recovery can be enhanced obviously, which can be ascribed to the synergetic effect of AOS and VES. The synergetic adsorption of AOS and VES in the oil/water interface can greatly decrease IFT and make the emulsification more easily. Therefore, the more residual oil can be flooded out more easily.

The relationship between incremental oil recovery and water separation ratio with different AOS concentrations is shown in Figure 8 (a), and the relationship between incremental oil recovery and interfacial tension is illustrated in Figure 8 (b). The two curves showed that the oil recovery can be enhanced by reducing interfacial tension or strengthen emulsion stability. It is also illustrated that both ultra-low interfacial tension and emulsion stability are significant mechanisms for EOR.

#### Effect of recycling system slug size and recycling system slug type

To study the optimal recycling system slug size, five tests (No. 5 and 8–11) were conducted by increasing the recycling system slug size from 0.1 to 0.9 PV while keeping the VES and AOS concentration at 0.02 wt% and 0.04 wt%, respectively.

**Figure 9:**
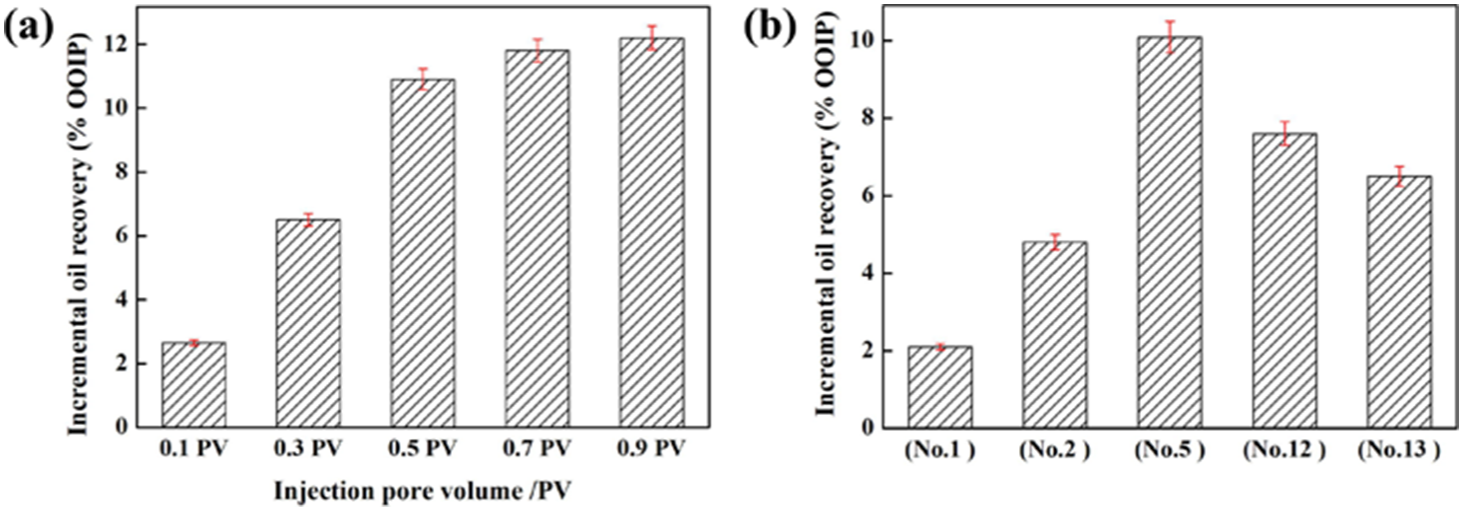
Effect of slug size and slug type on incremental oil recovery. (a) Effect of chemical slug size on incremental oil recovery; (b) effect of chemical slug type on incremental oil recovery.

Figure 9 (a) shows the effect of recycling system slug size on EOR. As shown in Figure 9, the incremental oil recovery shows a linear growth with the increment of recycling system slug size rangeing from 0.10 PV (2.65 OOIP%) to 0.50 PV (10.9 OOIP%). However, with a further increase of slug size, the incremental tendency of oil recovery slows, especially in the range of 0.70 PV (11.9 OOIP%) to 0.90 PV (12.1 OOIP%). The larger the slug size, the higher the incremental oil recovery. To make the process cost-effective, an optional slug size of 0.5 PV is chosen.

Figure 9 (b) shows that the final incremental recovery varies with the difference of recycling system slug type. Comparing the results of No. 1 (0.04 wt% AOS only), No. 2 (0.2 wt% VES only) and No. 5 (0.04 wt% AOS+0.2 wt% VES), it can be concluded that the highest incremental oil recovery was obtained in test No. 5 (10.9 OOIP%) when VES and AOS were injected simultaneously. The results prove that the synergy of VES and AOS is important for EOR in surfactant flooding. Figure 9 (b) also shows that, when 0.2 wt% VES and 0.04 wt% AOS are injected by turns and both the injection pore volumes are at 0.25 PV, their incremental oil recovery results are different and both lower than that of being injected simultaneously (10.9 OOIP% in No. 5, 7.6 OOIP% in No. 12, and 6.5 OOIP% in No. 13). The difference is scribed to the mixing degree of flowback fluid and AOS. When the AOS slug is injected first, it is easily washed out by the subsequent VES slugs because its low concentration and high interfacial tension. Therefore, less residual oil can be produced with this injection type.

#### Cumulative oil recovery of recycling system

**Figure 10:**
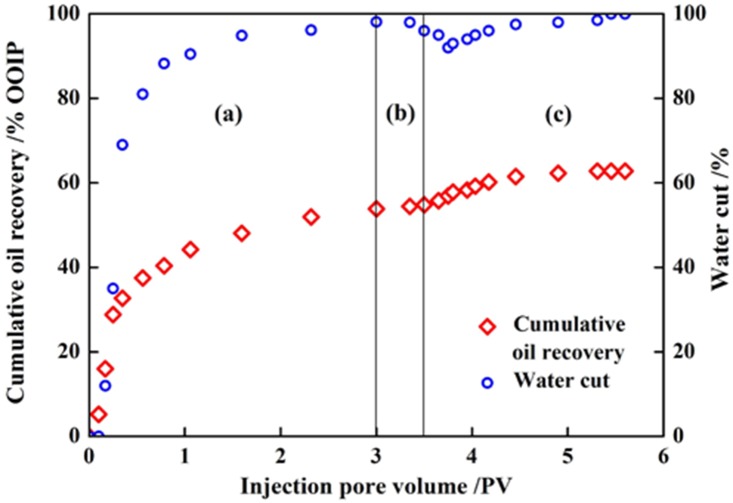
Cumulative oil recovery and water cut plots. Recycling system include 0.2 wt % VES and 0.04 wt % AOS, (a) initial water flooding; (b) recycling system flooding; (c) subsequent water flooding.

The cumulative oil recovery and water cut curves for core plug No. 5 with the injection of three slugs (initial water flooding slug, recycling system flooding slug, and subsequent water flooding slug) are shown in Figure 10. The VES/AOS flooding system consists of 0.2 wt% VES and 0.04 wt% AOS. It shows that there is a sudden water cut drop in the recycling system slug injection and subsequent water flooding. It also can be seen that an abrupt drop of water cut is accompanied by an incremental of oil recovery, a higher peak value of water cut drop results in a higher cumulative oil recovery. The increment of oil recovery is 10.9 OOIP% which indicates the recycling system’s better performance on EOR.

### Proposed mechanisms of reutilization of clear fracturing flowback fluids

**Figure 11:**
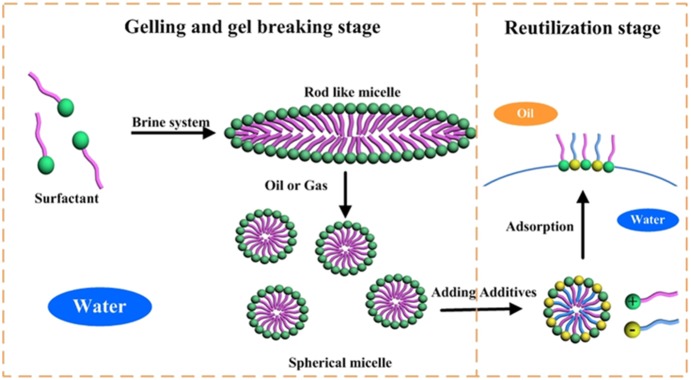
Proposed mechanism diagram of reutilization of clear fracturing flowback fluids.

The mechanisms of reutilization of fracturing flowback fluids are shown in Figure 11, which shows the process of gelling, gel breaking and reutilization of flowback fluids on chemical flooding after fracturing process.

The clear fracturing fluid is composed of viscoelastic surfactants. In aqueous solution, these molecules can assemble to form spherical micelles in an effort to separate their apolar regions from contacting aqueous phase. When dissolved in brine solution with a group of counter-anions [44] a group of surfactants can form rod-like micelles whose geometry is similar to that of polymer. The rod-like micelles entangled with each other to form the viscoelastic system, reported by Cates and Hoffmann [45]–[47].

When organic and other hydrophobic substances such as oil or gas [48], are dissolved in the micelles, the rod-shaped micelles are swelled become smaller spherical micelles again, leading to resultant loss in fluid viscosity. Since no inter breaker is added during the process of gel breaking stage. There is no damage to VES molecules, which provides solid foundation for the reutilization stage later.

The mechanism of reutilization of flowback fluid for surfactant flooding is illustrated in the right part of Figure 11. In the oil/water system, surfactants (VES, a cationic surfactant) tend to move to the oil/water interface with oil-soluble hydrocarbon chain towards oil phase and water-soluble group towards water phase. The adsorption of VES on the interface contributes to the decrease of IFT. With the addition of AOS (an anionic surfactant), more surfactants tend to move to the surface due to the strong electrostatic attraction between VES and AOS on the interface. The strong electrostatic attraction which is known as synergetic effect leads to the adsorption of more surfactant molecules on the oil/water interface, resulting in the appearance of lowest IFT and better emulsification ability in mixed catanionic systems, which is beneficial for EOR.

## Conclusions

In the present study, the reutilization of clear fracturing flowback fluids in surfactant flooding was studied initially. From what we have obtained above, the following conclusions can be drawn.

1. The synergetic effect between VES and AOS for reducing oil/water IFT and emulsifying oil is obvious when 0.2 wt% VES and 0.04 wt% AOS are simultaneously used. The IFT can be reduced by 2 orders of magnitude, the oil can be emulsified and dispersed more easily, and the emulsion stability is strengthened due to the synergetic adsorption of AOS. AOS has an obvious effect on the quality of recycling system while with little dosages ranging from 0.02 wt% to 0.08 wt%, which indicates that it is rather cost-effective compared with other treatment measures.

2. A larger VES/AOS slug size contributes to a higher incremental oil recovery, the optimal slug size (0.50–0.70 PV in this study) when taking expense factor into consideration. Besides, the highest incremental oil recovery is obtained when 0.2 wt% VES and 0.04 wt% AOS are injected simultaneously.

3. What we invested in this work verifies that reclaiming clear fracturing flowback fluids after fracturing operation and reuse it in surfactant flooding might have less effect on environment and be more economical.

## Supporting Information

File S1 Capillary number model.
(DOC)
