# Supplementary material for: Study on the Reutilization of Clear Fracturing Flowback Fluids in Surfactant Flooding with Additives for Enhanced Oil Recovery (EOR)
Source: PLoS One. 2014 Nov 19;9(11):e113723. doi: 10.1371/journal.pone.0113723 (PMC4237460; doi:10.1371/journal.pone.0113723)
Supplement: File S1 — Capillary number model. (DOC) [file pone.0113723.s001.doc]

**Supporting Information**

Study on the Reutilization of Clear Fracturing Flowback Fluids in Surfactant Flooding with Additives for Enhanced Oil Recovery (EOR)

Caili Dai^1^, Kai Wang^1,2^, Yifei Liu^1^, Jichao Fang^1^, Mingwei Zhao^1*^

*1 China University of Petroleum (East China), Qingdao, Shandong, 266580, People’s Republic of China*

*2 China National Offshore Oil Corporation Research Institute, Beijing, 100028, People’s Republic of China*

*Mingwei Zhao: Tel:+86-532-86981183 Fax:+86-532-86981161

E-mail: zhaomingwei@upc.edu.cn


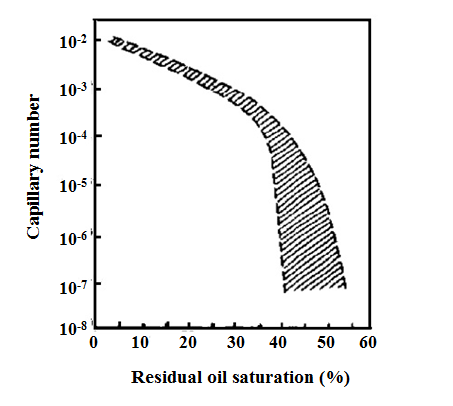


**Figure S1. The relationship between residual oil saturation and capillary number**

In surfactant or alkaline flooding systems, capillary number is a critical dimensionless number to the residual oil saturation of reservoir as shown in Figure S1. It is defined as the ratio of viscous force to capillary force of displacing phase and its formula is shown as following:

$$N_{c}=\frac{vu_{w}}{\sigma_{wo}}$$

Where, *N*_c_ is capillary number (no units); *v* is displacement velocity (m/s); *σ* is the oil/water interfacial tension (mN/m)；*u*_w_ is viscosity of displacing phase (mPa/s).

As is known, the increase of capillary number to a certain magnitudes contributes a lot to the reduction of residual oil saturation which can be as low as 20 %. The capillary number formula showed that the *N*_c_ will be significantly increased if the oil/water IFT can be reduced by 2 or 3 orders of magnitude. So, the reduction of IFT as low as possible is very important to the final oil recovery, which is generally decisive factor in the surfactant flooding.
